# Tibial Plateau Fracture: Implementing the Modified Frosch Approach for Dual-Benefit Achievement

**DOI:** 10.7759/cureus.77863

**Published:** 2025-01-23

**Authors:** Muhammad Firdaus Latip, Syed Syafiq, Ahmad Faiz Mohamad Khalil, Mohd Hisam Muhamad Ariffin

**Affiliations:** 1 Department of Orthopedics and Traumatology, Universiti Kebangsaan Malaysia Medical Center, Kuala Lumpur, MYS; 2 Department of Orthopedics and Traumatology, Hospital Canselor Tuanku Muhriz, Universiti Kebangsaan Malaysia (UKM), Kuala Lumpur, MYS

**Keywords:** extended schatzker, frosch approach, lateral tibial plateau, luo’s three column, two-column tibial plateau fracture

## Abstract

The increasing prevalence of tibial plateau fractures has led more orthopedic surgeons to focus on effective reduction and stabilization, which requires thorough preoperative assessment. The advent of computed tomography (CT) scans and three-dimensional reconstruction has revolutionized fracture analysis, enabled precise delineation, and improved surgical planning.

Historically, the tibial plateau classification based on 2D radiographs was the standard. However, the use of CT imaging has refined this with advancements in imaging, which categorizes fractures based on anatomical columns and cortical involvement. Recently, the classification has also included landmarks like the fibular tubercle and superficial medial collateral ligament, providing a more comprehensive quadrant-based classification.

In practice, we utilized a single-incision approach to address anterolateral and posterolateral fragments simultaneously. This technique is recognized for its safety, minimizing risks to vital structures like the peroneal nerve and arteries. By preserving ligament integrity and neurovascular structures, tailored surgical approaches can optimize outcomes.

Integrating advanced imaging techniques and refined classifications has significantly improved the management of tibial plateau fractures. These advancements allow surgeons to plan and execute surgeries with greater precision, enhancing patient outcomes. As the field evolves, ongoing research and innovation will likely lead to even more effective strategies, underscoring the importance of staying current with the latest orthopedic developments for optimal patient care.

## Introduction

The management of tibial plateau fractures has evolved significantly due to advancements in surgical techniques and imaging modalities. With the increasing incidence of these fractures, achieving optimal reduction and stability has become essential for improving clinical outcomes. Preoperative assessment of fracture morphology has become a key determinant in selecting the most appropriate surgical approach. The advent of computed tomography (CT) scans with 3D reconstruction capabilities has revolutionized the ability to precisely characterize these fractures, refining surgical planning.

Historically, the Schatzker classification system relied on 2D radiographs to guide treatment strategies for tibial plateau fractures. However, with the integration of CT imaging, a paradigm shift in fracture classification and surgical planning occurred. Luo et al. introduced the three-column theory, which categorizes fractures into anterolateral, anteromedial, and posterior columns, providing a more nuanced understanding of fracture patterns and facilitating tailored surgical interventions [1]. Recognizing the complexity of coronal plane fractures, Schatzker and Kfuri revised the classical classification, incorporating anatomical landmarks such as the lateral tubercle of the fibula and the superficial medial collateral ligament [2]. These virtual equators divide the tibial plateau into four quadrants, anterolateral, anteromedial, posterolateral, and posteromedial, each accessible through specific surgical approaches designed to preserve surrounding ligaments and neurovascular structures.

This case report highlights the use of the modified Frosch approach to address both anterolateral and posterolateral fragments simultaneously in a tibial plateau fracture. The approach is noted for its safety profile, minimizing risks to vital structures such as the common peroneal nerve, popliteal artery, and anterior tibial artery.

## Case presentation

**Figure 1:**
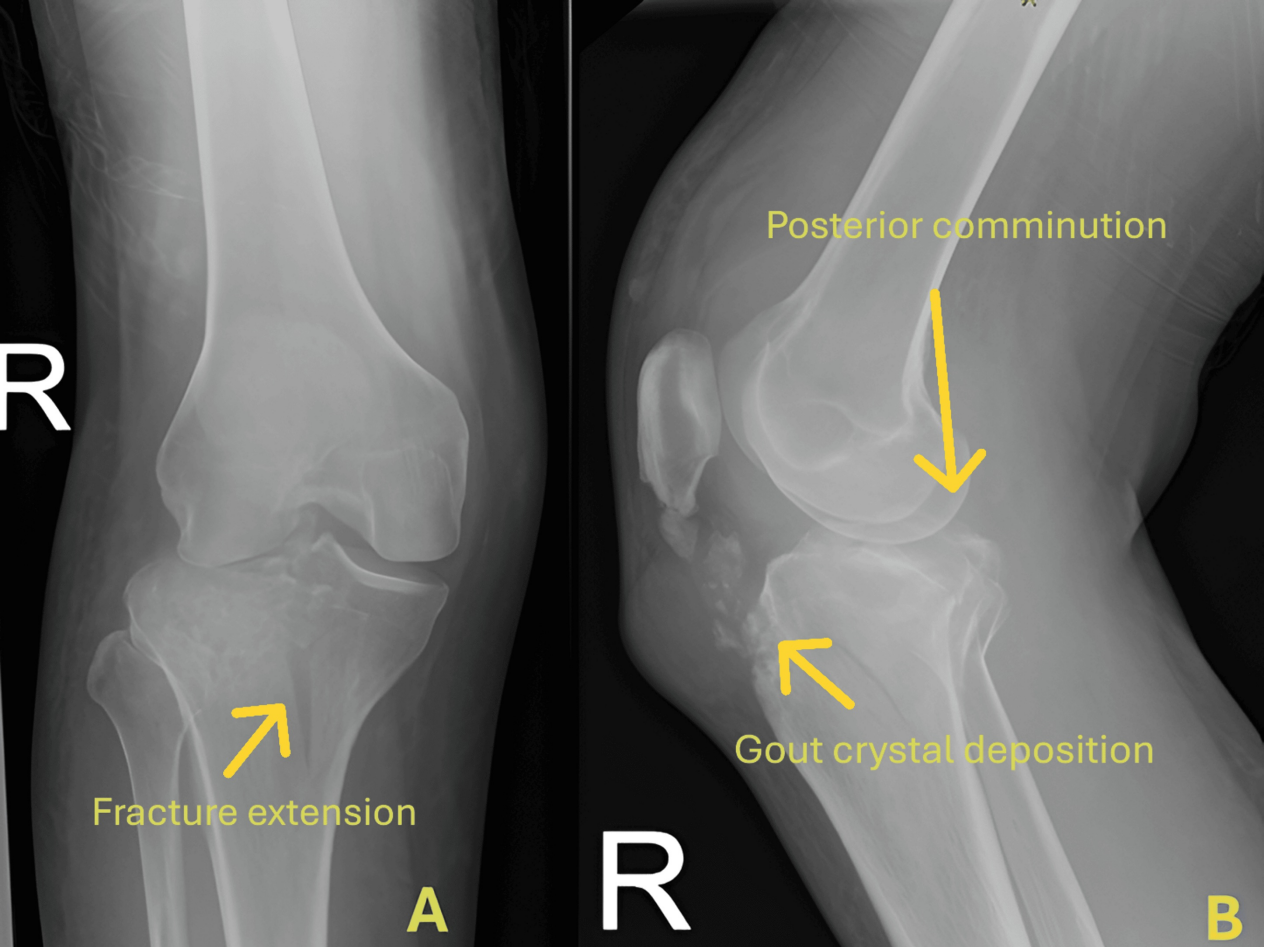
Radiograph showing (A) lateral tibial plateau depression with metaphyseal-diaphyseal extension and (B) posterior fragments. Calcification over the patella tendon is due to chronic gouty arthritis

A 39-year-old male patient presented to the emergency department with significant swelling in his right knee following a motor vehicle accident. He tried to avoid an oncoming car but landed heavily on his right knee, making it impossible for him to walk due to intense pain. On examination, there was marked swelling and tenderness over the upper tibia. The neurovascular assessment was unremarkable, and the knee compartment was soft, but movement was limited by pain. A radiograph revealed a lateral tibial plateau fracture extending into the metaphysis-diaphysis junction, classified as Schatzker VI (Figure 1). The patient has a history of gouty arthritis, currently treated with allopurinol, though it remains poorly controlled with recurrent acute flares. Radiographic findings also suggest calcification along the patellar tendon, which may indicate the presence of gouty tophi.

**Figure 2:**
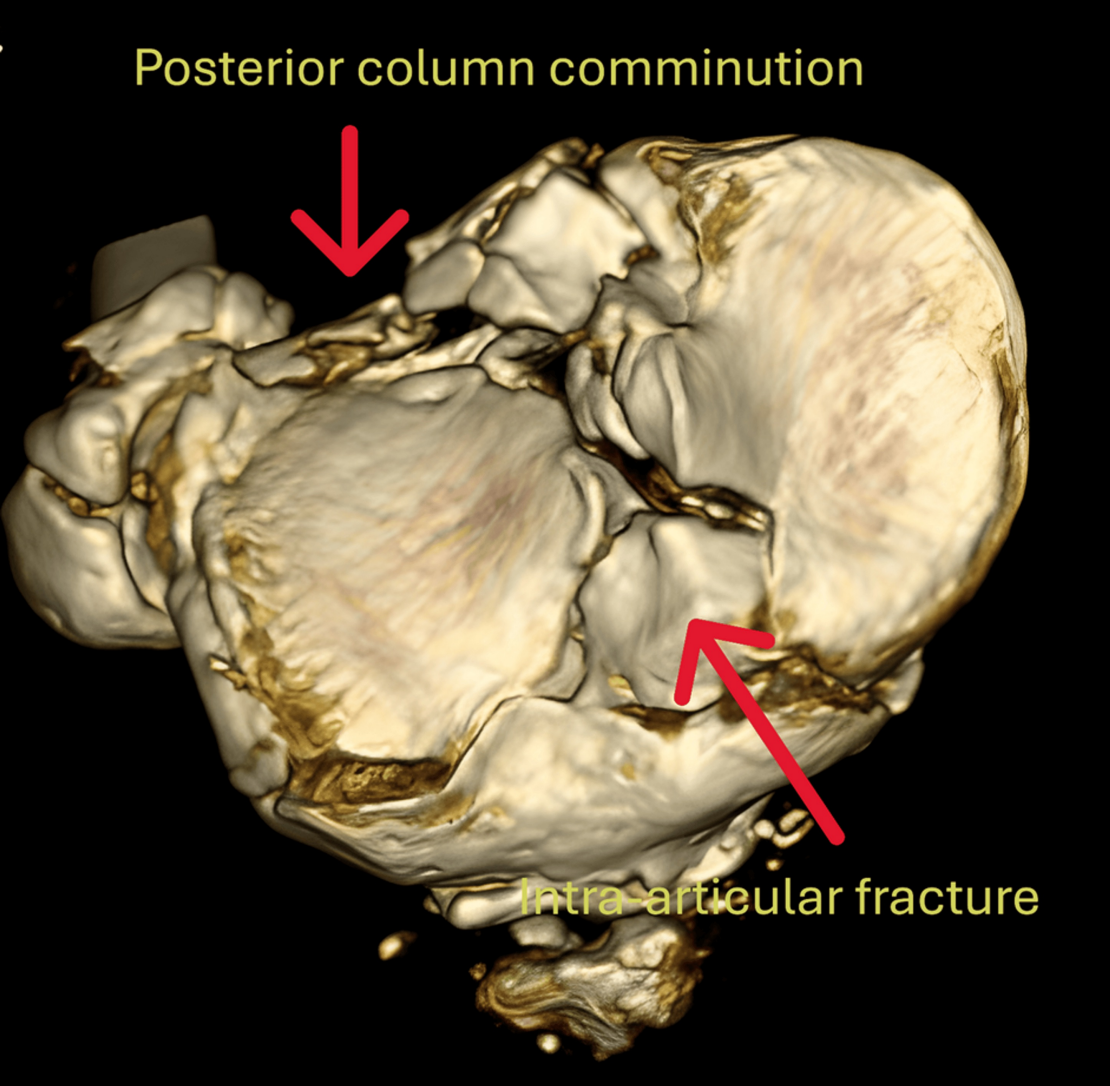
Axial 3D CT scan revealing posterior comminution with intra-articular fracture of the tibial plateau 3D CT: three-dimensional computed tomography

The early intervention included cryotherapy and calcaneal traction to manage soft-tissue swelling. CT imaging with three-dimensional (3D) reconstruction revealed a fracture involving the anterolateral and posterolateral columns, with significant intra-articular displacement (Figure 2).

**Figure 3:**
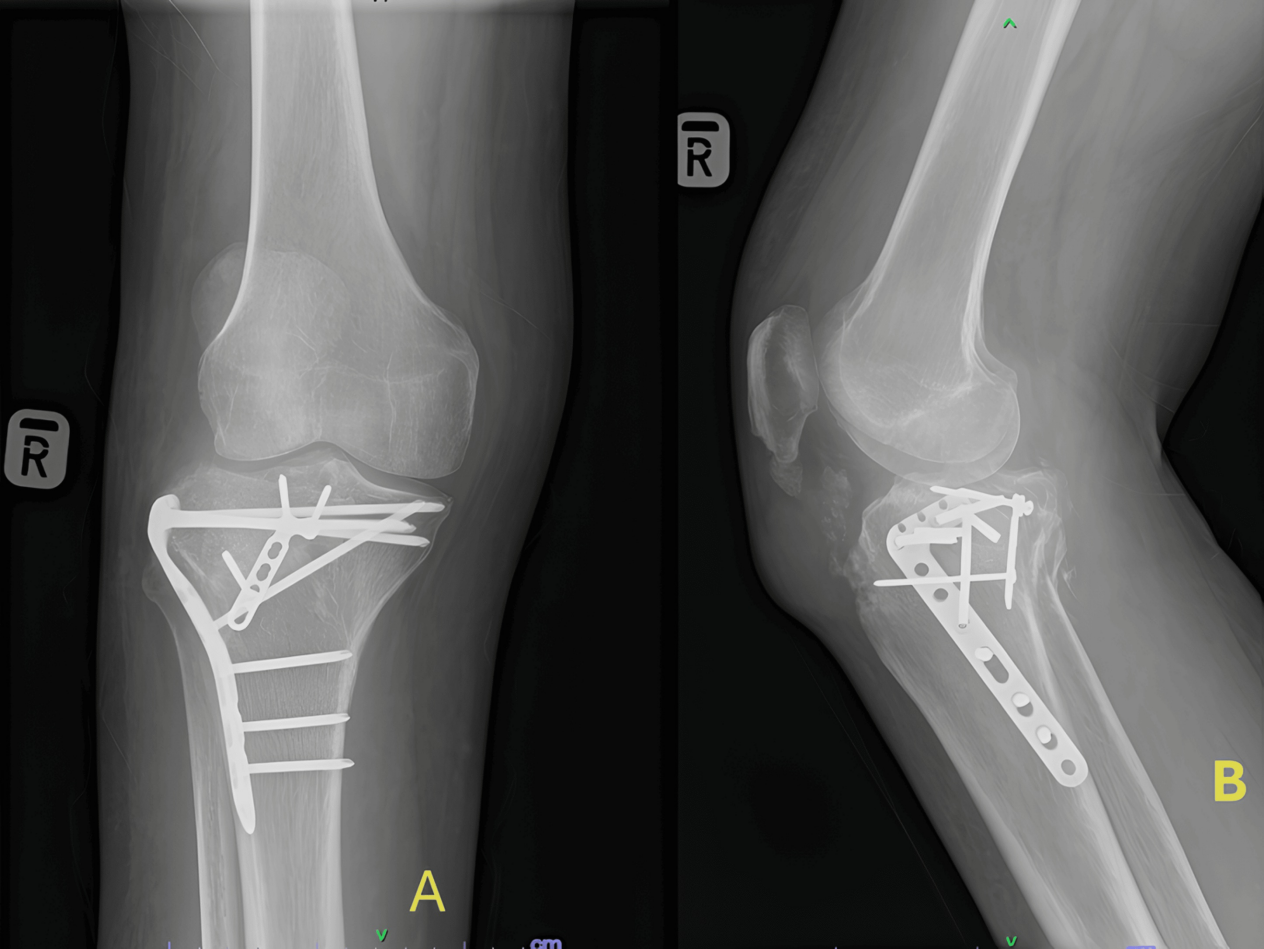
Six-month postoperative radiograph. (A) AP view showing restored congruent knee joint. (B) Lateral view showing that the posterior column is maintained with the posterior buttress AP: anteroposterior

The patient underwent open reduction and internal fixation using buttress plates via the modified Frosch approach [3,4]. The intra-articular fragments were realigned, and stability was restored using a six-hole anterolateral proximal tibia 4.5-mm locking compression plate (LCP) and a three-hole 3.5-mm metatarsal LCP as posterolateral buttress from DePuy Synthes (Synthes SDN BHD, Petaling Jaya, Malaysia). Three months later, the patient regained full range of motion and was able to bear weight while ambulating. The radiograph showed good fracture reduction and congruent knee joint restoration. However, the lateral tibial plateau is outside the lateral femoral condyle line, which may cause the patient to have post-traumatic osteoarthritis later (Figure 3).

**Figure 4:**
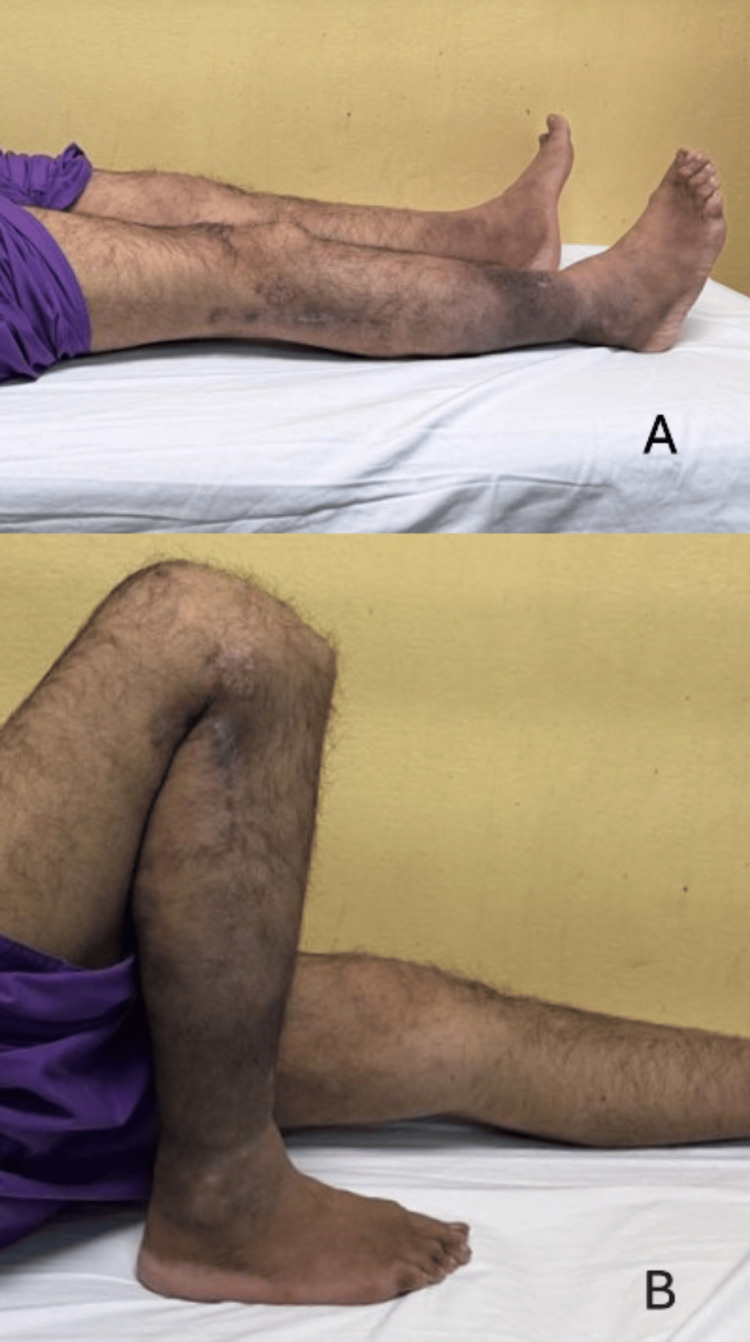
Six-month postoperative picture showing (A) good extension of the right knee and (B) full flexion of the right knee. The surgical scar healed well

The patient achieved satisfactory right knee function six months after the operation with a full range of motion and was able to climb stairs without any difficulty (Figure 4).

## Discussion

Utilizing the modified Schatzker classification with 3D CT scans of the knee joint, we can treat tibial plateau fractures by addressing the affected columns: anterior, posterior, medial, and lateral. This approach ensures optimal fracture stabilization and minimizes the risk of early post-traumatic osteoarthritis, adhering to the principle of periarticular fracture fixation: absolute stability, emphasizing the importance of achieving absolute stability.

In our case, the modified Frosch approach was used to address the anterolateral and posterolateral fragments simultaneously. This approach is considered safe, minimizing the risk of injury to vital structures such as the common peroneal nerve, popliteal artery, and anterior tibial artery. A 12-cm S-shaped skin incision is made along the lateral aspect of the knee, centered over the head of the fibula. The incision extends proximally, curving posteriorly to the lateral femoral condyle, and reaches distally midway between the tibial crest and the fibula. The common peroneal nerve is carefully identified and isolated from the posterior aspect of the biceps femoris tendon insertion and is safeguarded with a vessel loop. Structures in the popliteal fossa are meticulously protected. It is noted that the popliteal artery branches to form the anterior tibial artery, which passes anteriorly through the interosseous membrane between the tibia and the fibula approximately 5 cm distal to the knee joint. This anatomical detail limits posterior dissection to minimize the risk of injury to the anterior tibial artery.

**Figure 5:**
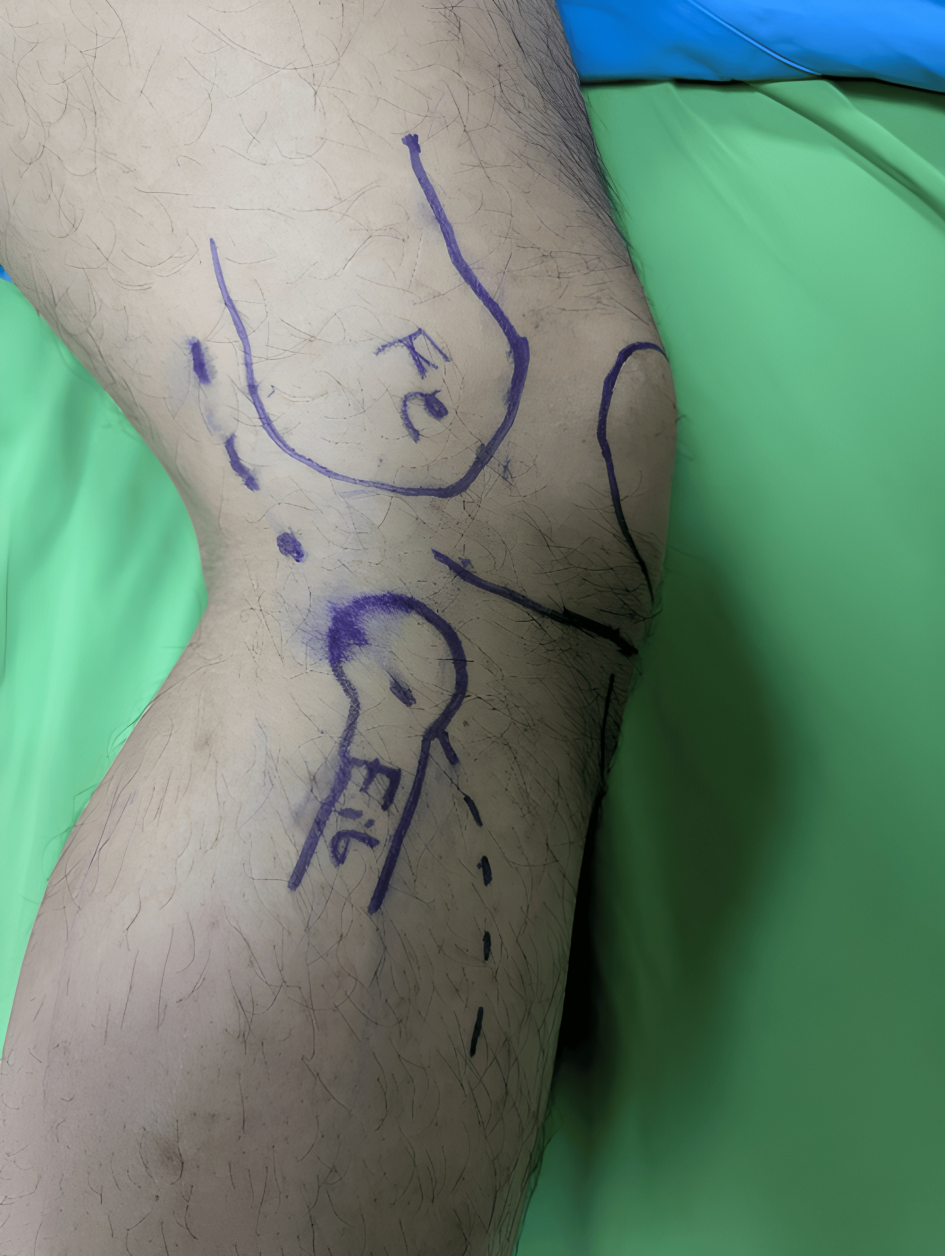
An anatomical landmark for the approach. The incision is centered at the head of the fibula, which extends proximally following the posterior border of the femur and distally between the fibula and the tibial crest forming a lazy S incision

**Figure 6:**
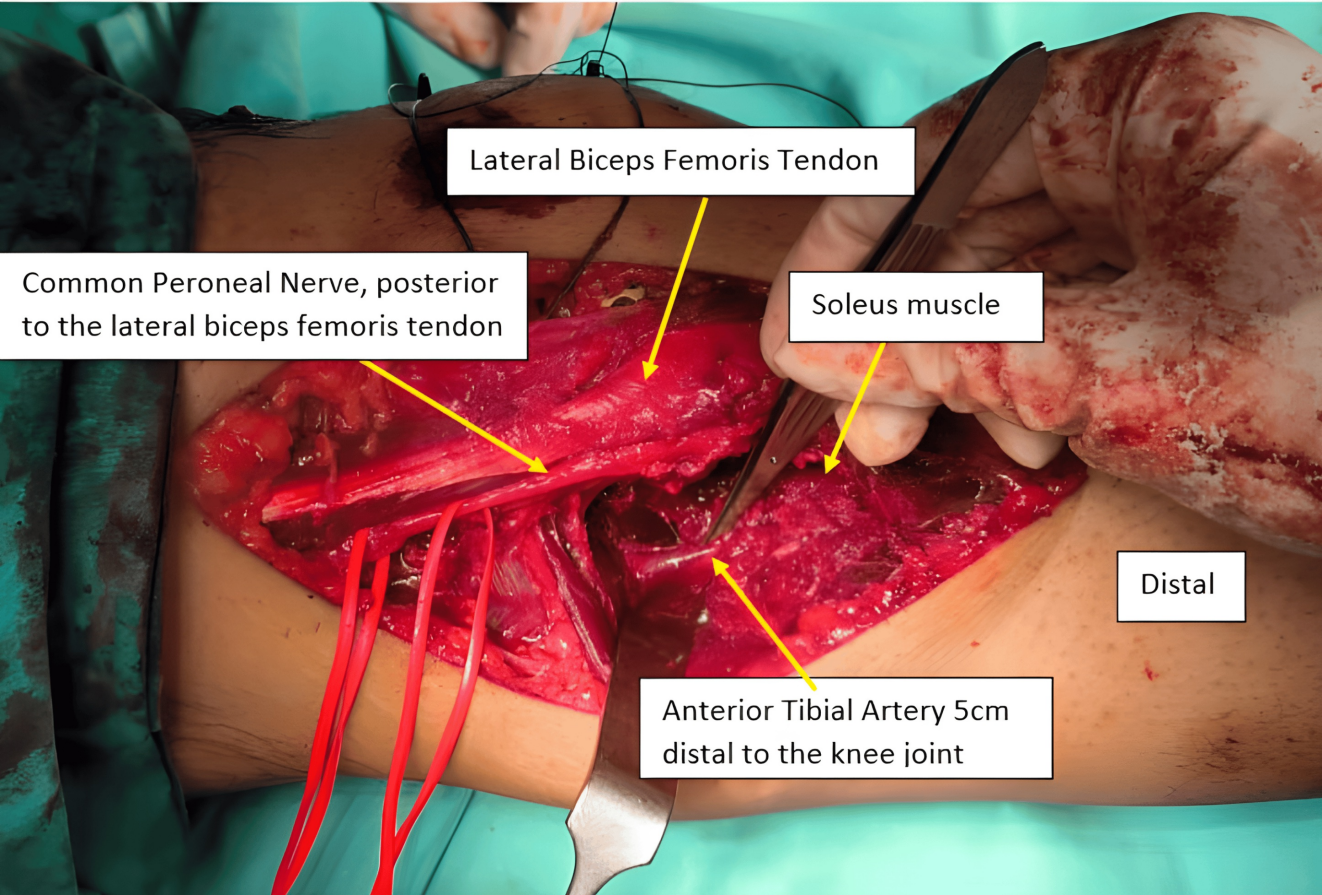
The common peroneal nerve is identified posterior to the lateral biceps femoris tendon, and the anterior tibial artery is seen 5 cm distal to the knee joint

The modified Frosch approach presents numerous advantages, including the requirement of a singular incision, the ability to easily adjust to a floppy lateral position [5], and the elimination of the need for fibula osteotomy. These factors collectively contribute to a reduction in operative time. However, some limitations include the potential risk of common peroneal nerve injury and restricted exposure to posterior fragments, which may limit implant size options (Figures 5, 6).

## Conclusions

The management of tibial plateau fractures has significantly advanced in response to their increasing incidence. The evolution of surgical techniques and the introduction of CT imaging with 3D reconstruction capabilities have greatly improved the preoperative assessment of fracture morphology and the ability to plan surgical interventions. These advancements have allowed surgeons to develop tailored approaches that preserve the integrity of surrounding structures, ultimately improving patient outcomes.

As demonstrated in this case, the use of the modified Frosch approach enabled the successful treatment of a complex tibial plateau fracture while minimizing risks to vital structures. The evolving landscape of fracture management, supported by innovations in imaging and surgical techniques, continues to enhance outcomes for patients with tibial plateau fractures. Future research and technological advancements promise further refinement of treatment strategies, ensuring continued progress in the field of orthopedic trauma.
